# Intelligent diagnosis system based on artificial intelligence models for predicting freezing of gait in Parkinson’s disease

**DOI:** 10.3389/fmed.2024.1418684

**Published:** 2024-06-20

**Authors:** Abdullah H. Al-Nefaie, Theyazn H. H. Aldhyani, Nesren Farhah, Deepika Koundal

**Affiliations:** ^1^King Salman Center for Disability Research, Riyadh, Saudi Arabia; ^2^Department of Quantitative Methods, School of Business, King Faisal University, Al-Ahsa, Saudi Arabia; ^3^Applied College in Abqaiq, King Faisal University, Al-Ahsa, Saudi Arabia; ^4^Department of Health Informatics, College of Health Sciences, Saudi Electronic University, Riyadh, Saudi Arabia; ^5^School of Computer Science, University of Petroleum and Energy Studies, Dehradun, India

**Keywords:** freezing, Parkinson’s, gait, machine leaning, prediction, classification, transformers models

## Abstract

**Introduction:**

Freezing of gait (FoG) is a significant issue for those with Parkinson’s disease (PD) since it is a primary contributor to falls and is linked to a poor superiority of life. The underlying apparatus is still not understood; however, it is postulated that it is associated with cognitive disorders, namely impairments in executive and visuospatial functions. During episodes of FoG, patients may experience the risk of falling, which significantly effects their quality of life.

**Methods:**

This research aims to systematically evaluate the effectiveness of machine learning approaches in accurately predicting a FoG event before it occurs. The system was tested using a dataset collected from the Kaggle repository and comprises 3D accelerometer data collected from the lower backs of people who suffer from episodes of FoG, a severe indication frequently realized in persons with Parkinson’s disease. Data were acquired by measuring acceleration from 65 patients and 20 healthy senior adults while they engaged in simulated daily life tasks. Of the total participants, 45 exhibited indications of FoG. This research utilizes seven machine learning methods, namely the decision tree, random forest, Knearest neighbors algorithm, LightGBM, and CatBoost models. The Gated Recurrent Unit (GRU)-Transformers and Longterm Recurrent Convolutional Networks (LRCN) models were applied to predict FoG. The construction and model parameters were planned to enhance performance by mitigating computational difficulty and evaluation duration.

**Results:**

The decision tree exhibited exceptional performance, achieving sensitivity rates of 91% in terms of accuracy, precision, recall, and F1- score metrics for the FoG, transition, and normal activity classes, respectively. It has been noted that the system has the capacity to anticipate FoG objectively and precisely. This system will be instrumental in advancing consideration in furthering the comprehension and handling of FoG.

## 1 Introduction

Parkinson’s disease (PD) is a degenerative neurological sickness that disturbs a large number of individuals (1). Freezing of gait (FoG) and the subsequent increased risk of falls are the primary disabling issues for a noteworthy figure of individuals with PD (2). There are presently few options for pharmacological therapies. Several tools and wearable devices that make available treatments, like rhythmical cueing and step-synchronized vibratory cueing, demonstrate good concert and results (3). Efficient treatment of FoG is now being investigated via examination on FoG recognition and prediction.

**Figure 1:**
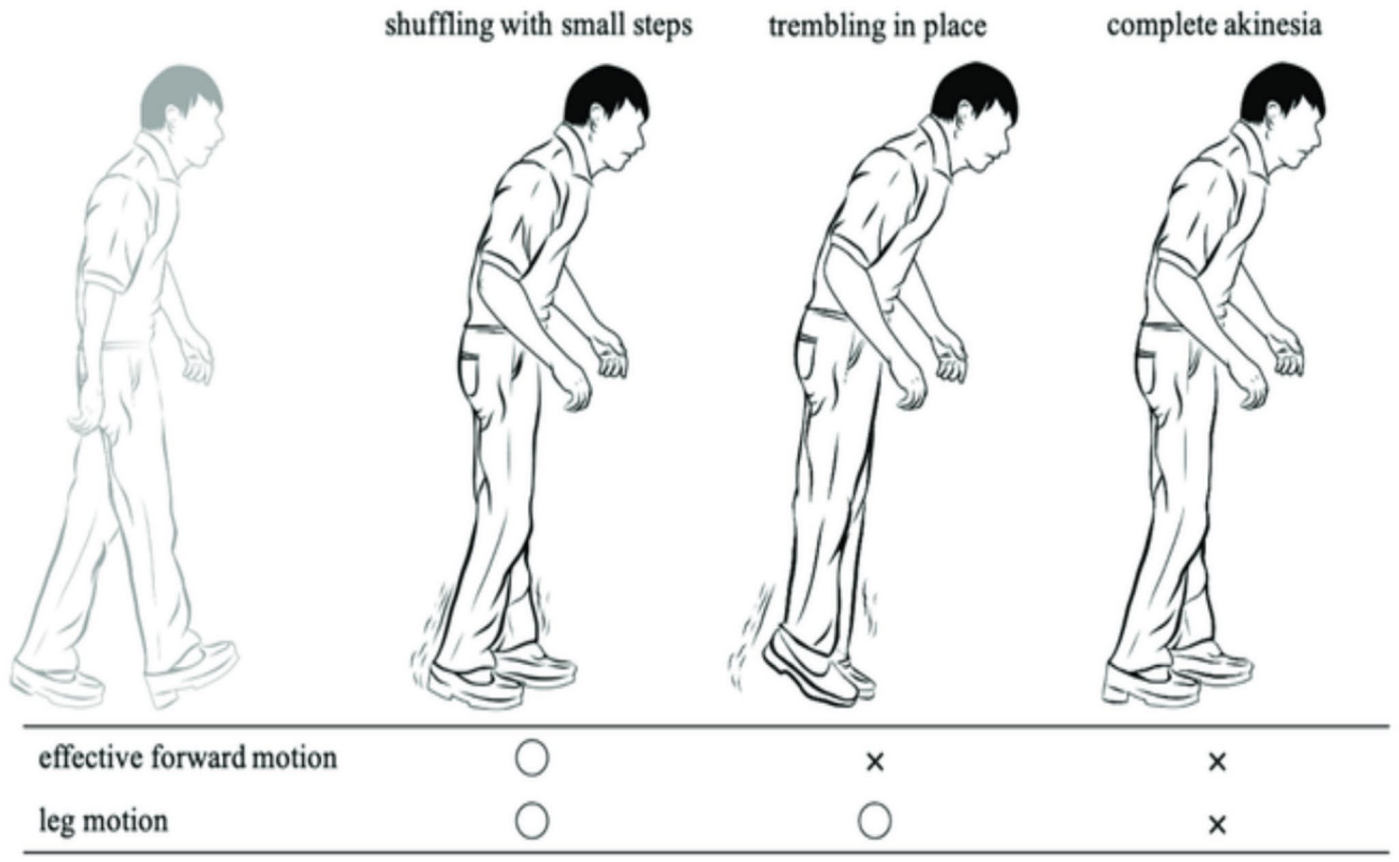
FoG sporadic walking.

FoG is a sporadic walking problem characterized by sudden interruptions in stride or a significant decrease in forward movement of the feet (4). It greatly impacts quality of life and increases the likelihood of reductions and breakages in individuals with PD (2, 5). These symptoms may disrupt patients’ everyday activities, jeopardize their mental well-being, and lead to a weakening in their superiority of life. Approximately half of individuals with PD have encountered signs of FoG, which is the primary factor leading to falls (6–8). FoG is characterized as a temporary and intermittent inability or noteworthy reduction in the advancing motion of the feet, even when there is a desire to walk. In their study, Schaafsma et al. (9) categorized FoG into five distinct subtypes: start hesitation, turn hesitation, hesitation in confined spaces, hesitation toward a specific goal, and hesitation in wide spaces. Typically, FoG is linked to a particular sensation of “the feet being adhered to the ground” (10). FoG is influenced by surroundings, drugs, and anxiety, which might impact its frequency and duration (11). FoG is often considered to be a characteristic of akinesia, which is a severe type of bradykinesia (12). FoG is characterized by transient periods of immobility or the execution of very small steps while attempting to begin walking or change direction (2). The state of FoG is significantly influenced by ambient cues, cognitive input, medicines, and anxiety (11, 13). It is more common to experience it at home rather than in a clinical environment, particularly in scenarios when there is full darkness or when there is a higher cognitive load, such as dual-tasking conditions (14–17). Figure 1 displays FoG sporadic walking.

FoG is a very debilitating condition often seen in individuals with PD. The symptoms often manifest in the later stages of the illness, with roughly 50% of all PD patients experiencing some indications and around 80% being significantly impacted (10, 18–20). Episodes of FoG often present as a sudden and temporary inability to initiate movement, often occurring while starting to walk, during making turns, or under stressful circumstances. During bouts of FoG, individuals with PD experience a phenomenon where they perceive their feet to be firmly stuck to the ground without any apparent cause (9). During episodes of FoG while walking, patients exhibit variations in their walking pattern and experience a significant decrease in the length of their steps. Additionally, they often display shaking in their legs (19, 20). The typical frequency range for normal gait steps, as measured by ankle sensors, is 0.5 to 3 Hz. However, FoG occurrences have a higher rate variety of 6 to 8 Hz (21–23).

Recent research has begun using machine leaning and deep learning for the resolution of automated categorization. Deep learning is a branch of artificial intelligence (AI) that utilizes algorithms having capability of mechanically extracting distinguishing features from information and data, such as signals acquired straight from sensors without any prior processing. Deep learning (DL) and machine learning (ML) have facilitated the creation of classifiers that cover the entire process and have demonstrated exceptional performance in various fields, including image processing, computer vision, medical information analysis, bioinformatics, natural language processing, logical reasoning, robotics, and control (24–27). Therefore, DL techniques have been used in human activity recognition (HAR) systems utilizing data collected from various light sensors (28, 29).

DL and ML methods have become more popular for detecting FoG in recent years, as seen by the employment of these techniques in several studies (30–34).The following are the most significant and noteworthy. Kim et al. (30) and Pepa et al. (32) introduced a novel sensing tool, namely a smartphone positioned in the pant pocket, as a more convenient method for monitoring patients with PD and detecting FoG. The researchers used a technique that relied on convolutional neural networks (CNN) to automatically extract distinctive characteristics from sensors integrated into an Android smartphone. The performance of the CNN classifier was compared to that of the random forest (RF) classifier, and the CNN classifier exhibited a sensitivity that was 20% greater than that of the RF classifier.

Approximately 7 to 10 million individuals worldwide are affected by PD, with a significant portion experiencing FoG. During an episode of FoG, a patient experiences a phenomenon where their feet get immobilized, making it impossible for them to go forward despite their efforts. FoG significantly impairs health-related quality of life, leading to depression, heightened fall risk, greater reliance on wheelchairs, and limited autonomy.

This study used a standardized dataset obtained from 65 participants, using a 3D accelerometer. The dataset has been categorized into four classes: Normal, Turn, Walking, and StartHesitation. Preprocessing methods were suggested to cleanse the dataset and address the issue of imbalanced classes. The output from the preprocessing approach was analyzed using several ML, deep learning and transformers modes to determine if the patients are experiencing FoG or are in a normal state. The primary contribution of this work is as follows:

1The initial system employed for the classification of FoG used a new dataset.

2In our research, we have categorized the dataset into four distinct classes namely Normal, Turn, Walking, and StartHesitation because the dataset did not have labels.

3Employed various of ML, deep learning, and transformer approaches to predict the occurrence of FoG in patients with PD, the system achieved 91% with respect to accuracy.

## 2 Background of the study

FoG is an indication often seen in people with PD. However, the fundamental mechanisms of FoG are not well understood. Patients with PD often report this symptom as a sensation of their feet being firmly adhered to the ground (34–37). Handojoseno et al. (38) utilized the wavelet factors of electroencephalogram (EEG) data as the input for the multilayer perceptron neural network and KNN technique. This method achieved a sensitivity of 87% and an accuracy of 73% in predicting the transition from walking to FoG. Delval et al. (39) used a multi-camera setup to capture the gait kinematics gestures of patients. Deep pointers were affixed to the patients’ bodies and recorded from various angles. Okuno et al. (40) utilized a plantar pressure measurement system of 1.92 m × 0.88 m for recording the walking patterns of patients by monitoring the weight exerted on their soles. While the sensors may all be used for FoG detection, the predominant method for FoG detection in community environments relies on inertial sensors.

Moore et al. (21) developed a portable monitoring apparatus and algorithm that used the occurrence features of vertical leg movement. This movement was measured using an accelerometer put on the left shank of 11 individuals with PD. The contributors’ ages ranged from 45 to 72 years. The contributors were trained to go through a series of interior passages, including a tight entryway, and three obstacles. This research took into account the specific effects of the levodopa/carbidopa drug combination throughout both the “on” and “off” periods. The researchers used a threshold-based method to identify FoG, achieving a FoG detection rate of 78% and an accuracy rate of 89%. Delval et al. (39) conducted research in which they induced FoG in patients and used a series of measurable indicators to identify the presence of FoG. They used a 3D motion-analysis device to capture video footage of 10 sick and 10 healthy people while they were on a treadmill. Indicators were affixed to the heels, toes, ankles, shoulders, and on the T10 vertebra. Obstacles were encountered due to special situations, causing the patients to be in an inactive state. The identification of FoG in that particular investigation relied on a combination of threshold and frequency investigation. Bachlin et al. (41) devised a FoG recognition architecture using three accelerometers and implementing Moore’s threshold-based algorithm (21). Upon detecting an episode of FoG, the device used a metronome to offer stimulation to the patient, aiding them in regaining their focus and stability. The system support resulted in improved gait for six out of eight individuals who had FoG. Azevedo et al. (41) Developed a FoG detector that included gait pattern analysis by using a solitary inertial sensor positioned on the lower extremity. Based on its findings, it determines that relying just on frequency-based analysis is insufficient for accurately identifying the occurrence of FoG. It is essential to not only detect but also forecast when a FoG event will take place. The authors used rhythm and tread data into their methodology to enhance the categorization process. In order to assess the walking patterns of individuals with PD, Jovicic et al. (42) developed a technique that utilizes inertial sensors placed on both lower legs to categorize different gait patterns. The system also distinguished between regular and pathological gait by utilizing an expert rule-based approach, based on data collected from 12 PD patients who walked over a convoluted course. A rule-based categorization approach was used for the identification and categorization of FoG. Pham et al. (43) introduced a FoG detection method that is not reliant on specific individuals. The uniqueness of this idea is in its ability to operate autonomously from the topic matter. An additional instance of a FoG recognition system that uses wearable accelerometers and video capture to categorize the occurrence is shown in the research conducted by Zach et al. (44). Their finding suggests that FoG may be detected with just one accelerometer placed in the lumbar area.

Pepa et al. (32) used soft computing approaches for FOG identification. A fuzzy method was created to integrate information pertaining to freeze index, energy, cadency fluctuation, and the derivative energy ratio. A building was constructed that relied on a smartphone as its foundation. Their findings demonstrated that, on average, the system exhibited a specificity of 92.33% and recall of 83.33% in classifying FoG events. Cole et al. (36) presented a method using dynamic neural networks (DNN) to accurately identify FoG. They gathered information from three accelerometers and an electromyographic shallow worn by patients and achieved favorable consequences in terms of detection. A noteworthy involvement of this study is the creation of a database documenting unscripted and unimpeded everyday activities of PD patients, including instances of FoG. Ahlrichs et al. (22) introduced a FoG detector that utilizes a single accelerometer worn at the waist and a recognizer based on SVM. They documented the performance of 20 people with PD engaging in pre-planned everyday tasks. Patients were required to be documented both when taking medicine and while not taking medication. Their findings demonstrated a precision rate of 98.7%.

Rodrıguez-Martın et al. (45) developed a ML method designed to identify episodes of FoG. Their preference for FoG detection was SVM. Their technique relies on a solitary 3D accelerometer positioned at the waist to identify FoG in real-world scenarios. A total of 21 individuals diagnosed with PD contributed in the research work. The patients were asked to execute two sets of pre-determined exercises during both their “off” and “on” times. These activities were associated with everyday existence. According to their research, the medicine had an impact on the patients’ motor reaction. Deep learning methods have been popular for detecting FoG in recent years, as seen by their frequent application in research (30, 34, 46–48). Kim et al. (30) used a novel sensing device, namely a smartphone positioned in the trouser pocket, to discover a more pragmatic approach for monitoring patients with PD and identifying FoG. The researchers used a technique that relied on CNN to automatically extract distinctive characteristics from sensors integrated into an Android smartphone. The performance of the CNN method was compared to that of the RF technique, and the CNN exhibited a sensitivity that was 20% better that of the RF classifier. Xia et al. (49) suggested a FoG detection method based on CNN to accomplish automated feature learning and classification for FoG. Bachlin et al. (41) conducted experiments that relied on the patient’s input and studies that did not need the patient’s involvement. The most favorable outcomes were documented in the patient-dependent experiments. Same researchers used DL to predict FoG and PD (50–53).

## 3 Materials and methods

**Figure 2:**
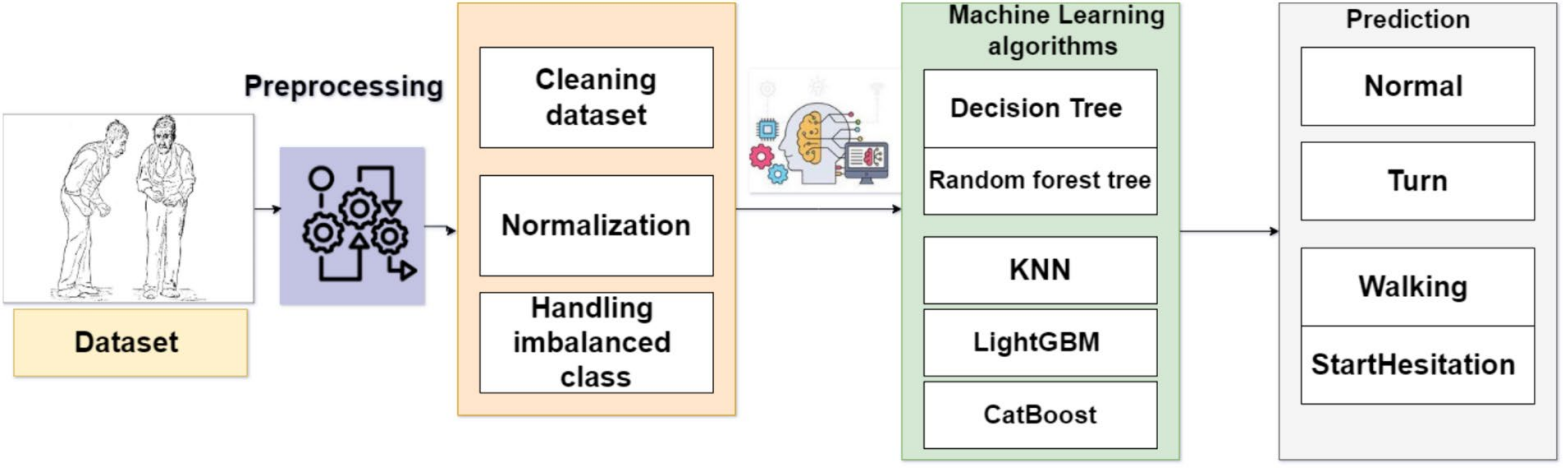
Framework of the system to predict FoG.

The proposed system aims to identify FoG, a distressing symptom that affects many individuals with PD. The proposed solution is built upon a machine learning models that have been trained using data obtained from a wearable 3D sensor device positioned on the lower end. Figure 2 displays the framework of the FoG system based on a machine learning approach.

### 3.1 Dataset

The dataset was obtained from the Kaggle repository and consists of 3D accelerometer data from the lower back of individuals experiencing bouts of FoG, a debilitating condition often seen in individuals with PD. FoG has a detrimental effect on the ability to walk, hindering movement and independence. The goal is to identify the initiation and termination of each freezing episode, as well as the presence of three specific kinds of FoG events: start hesitation, turning, and walking. The data series consists of three unique datasets, each obtained under separate circumstances: (1) The tDCS FoG (tdcsfog) dataset consists of data series obtained in a laboratory setting, where individuals underwent a FoG-provoking procedure; (2) The DeFOG dataset consists of data series that were obtained in the subject’s home as they conducted a FoG-provoking regimen; and (3) The daily living dataset consists of 1 week of uninterrupted 24/7 recordings from 65 people. Out of the total number of participants, 45 display symptoms of FoG and also have series in the DeFOG dataset. In contrast, the other 20 patients do not show any symptoms of FoG and do not have series in any other part of the data. Table 1 displays meta data, whereas the training dataset is presented in Table 2.

**Table 1:** Metadata of dcsfog and tdcsfog.

Features name	Description	Types of dataset
Visit		Int64
Medication		Int64
Time	A numerical value representing a discrete unit of time. The tdcsfog dataset records series at a frequency of 128 Hz, meaning there are 128 timesteps per second. On the other hand, the defog and daily series are recorded at a frequency of 100 Hz, resulting in 100 timesteps per second.	
AccV, AccML, AccAP	The lower-back sensor measures acceleration along three axes: vertical (V), mediolateral (ML), and anteroposterior (AP). The data is expressed in units.	Float64
Event	Class	Object

**Table 2:** features of dataset.

Features name	Description
Visit	Lab visits include an initial evaluation, two subsequent evaluations for distinct therapy phases, and a final evaluation for follow-up purposes.
Test	Test used
Medication	Subjects may have been either receiving or not receiving anti-parkinsonian medication throughout the recording.

### 3.2 Preprocessing approach

**Figure 3:**
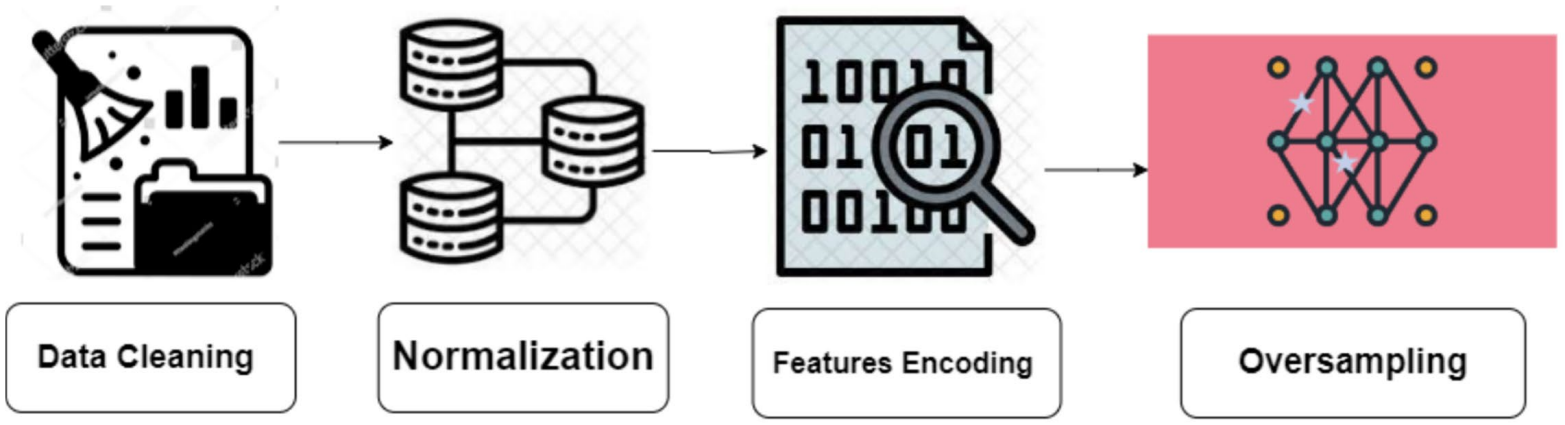
Preprocessing steps.

Data features engineering require the creation of new features or the transformation of existing features to enhance the effectiveness of a machine-learning model. Data preprocessing entails the extraction of pertinent information from unprocessed data and converting it into a format that is readily comprehensible by a model. The objective is to enhance the precision of the model by providing more significant and relevant data. The missing values in the dataset were removed from all features. We have combined DeFOG features, namely Time, AccV, AccML, and AccA, with the DeFOG-metadata for Subject, Visit, and Medication Condition. Figure 3 shows the preprocessing steps for the classification of FoG of PD patients.

#### 3.2.1 Normalization

Normalization is an essential preprocessing step for any machine-learning task. The process can be executed by either scaling or altering the initial data in order to equalize the influences of various characteristics in the data examples. In the present research work, we have standardized the input data to generate a representation among one and zero.


(1) $$x_{\mathrm{normalize}}=\frac{x-x_{\text{min}}}{x_{\text{max}}-x_{\text{min}}}$$

Where the *x* is training data, and $x_{\text{min}}$ is maximum value [1] and $x_{\text{min}}$ is minimum value [0].

#### 3.2.2 Handling imbalance classes

Unbalanced data raises to a condition where the representation of observations and samples among dissimilar classes is unequal, with one class dominating the dataset and the other classes having insufficient representation.

The synthetic minority oversampling strategy (SMOTE) is a resampling strategy used to address extremely imbalanced datasets by creating synthetic samples in the minority class, hence increasing its representation. SMOTE is effective in increasing the figure of minority class examples and achieving class balance. To mitigate the problem of overfitting, the synthetic production of fresh samples deviated from the increase procedure.

**Figure 4:**
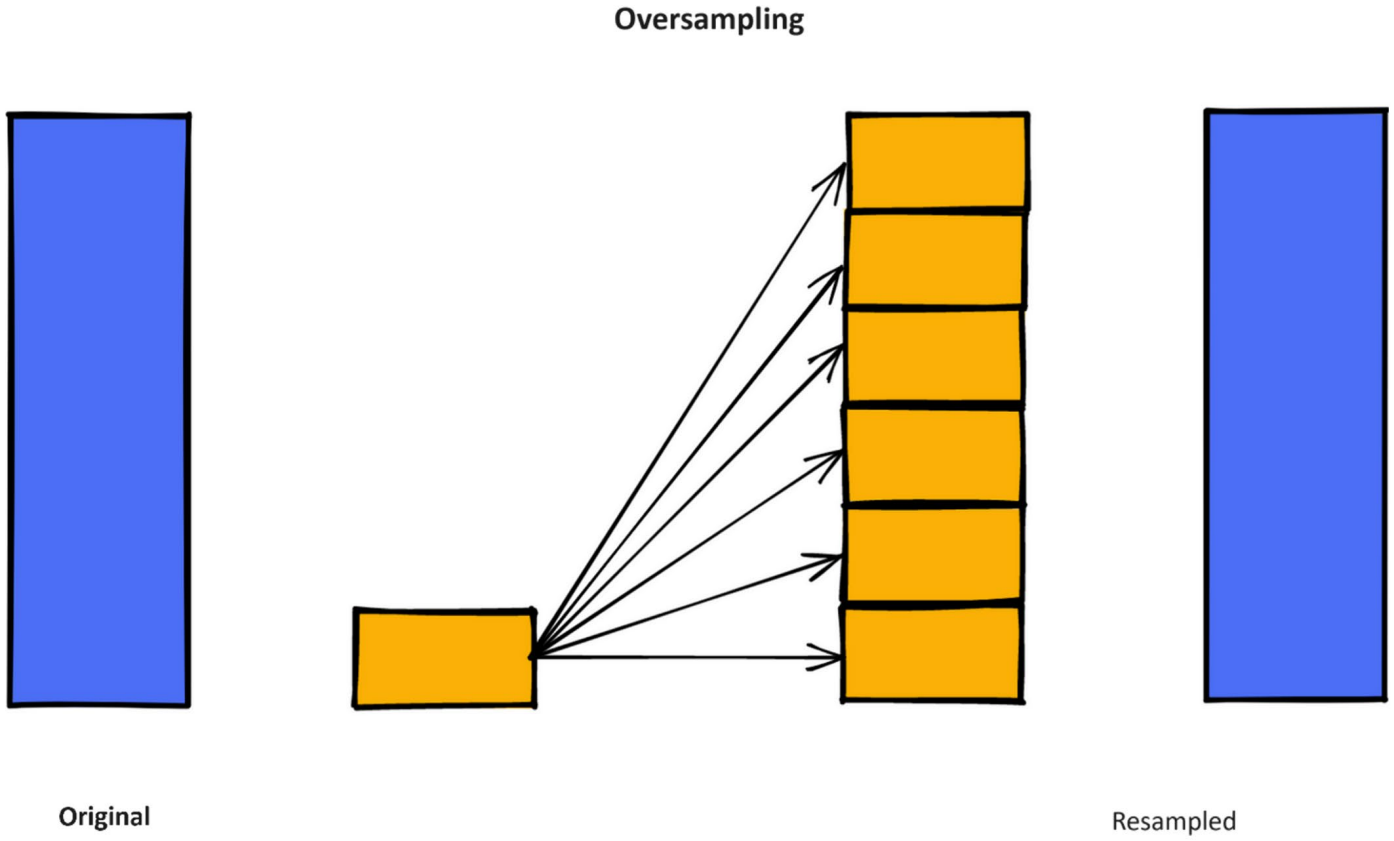
Working of SMOTE method.

The primary concept behindhand SMOTE technique is to create additional data samples in the minority class using interpolation between neighboring examples within this class (54). SMOTE enhances the amount of instances belonging to the minority class in an unbalanced dataset, thus improving the classifier’s ability to generalize well. Figure 4 shows the SMOTE method in practice.


(2) $$D_{\mathrm{new}}=D_{i}+\left({\hat{D}}_{j}-D_{i}\right)x\delta$$

The dataset $D_{\mathrm{new}}$ represents the ADHD dataset. $D_{i}$ consists of samples from the minority group, whereas ${\hat{D}}_{j}$ is a k-nearest neighbor of $D_{i}$ . Let δ represent a uniformly distributed random number between 0 and 1. We used the SMOTE technique to enhance the categorizing process.

**Figure 5:**
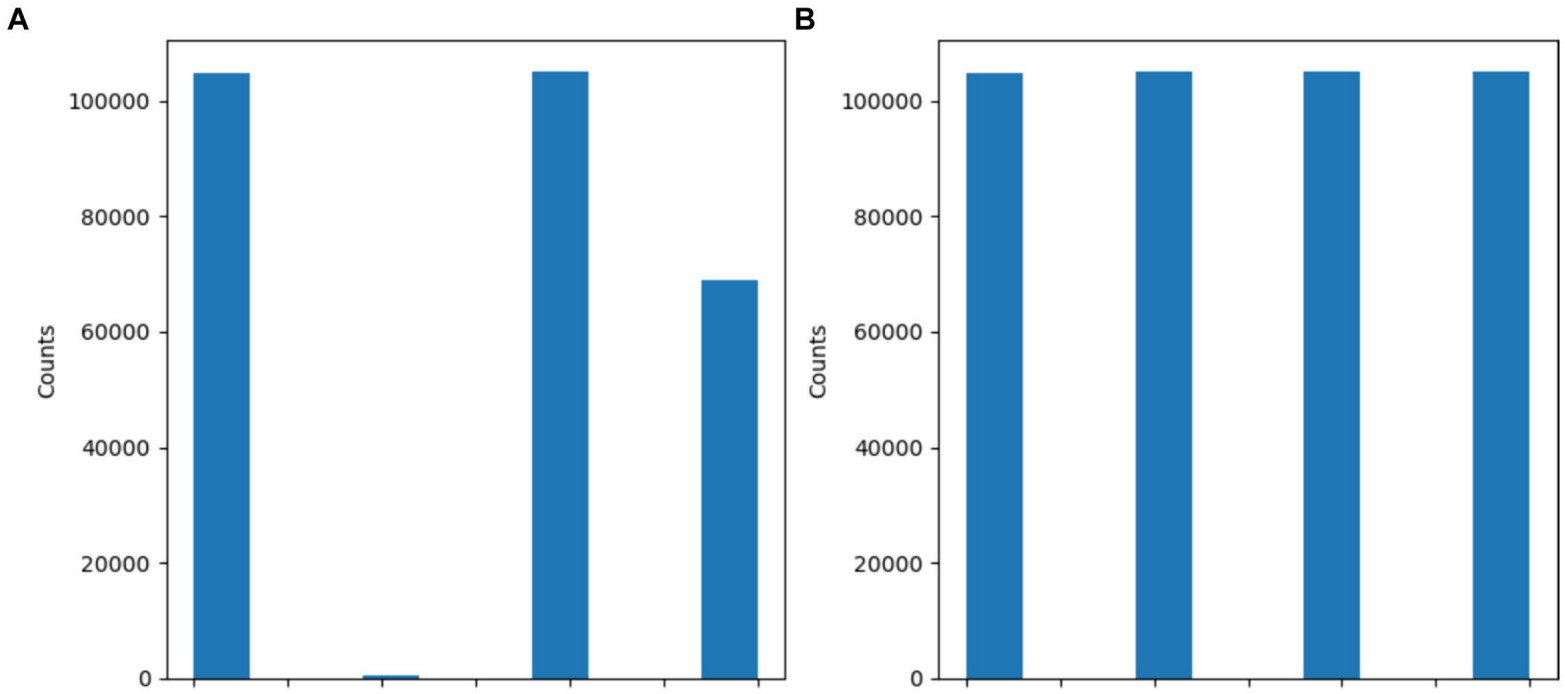
Results of SMOTE approach **(A)** before SMOTE **(B)** After SMOTE.

Figure 5 and Table 3 show the dataset before and after class distribution of the dataset using the SMOTE approach in the training dataset. The startHesitation class has less values (352); therefore, we have applied the SMOTE approach for handing this imbalance class to enhance the machine algorithms.

**Table 3:** Results of SMOTE approach.

Before the SMOTE	
Classes	Values
Normal	105,176
Turn	104,785
Walking	68,999
StartHesitation	352
After the SMOTE	
Normal	105,176
Turn	105,176
Walking	105,176
StartHesitation	104,785

### 3.3 Algorithms

#### 3.3.1 K-nearest neighbors

The KNN technique is a straightforward nonparametric approach that\ is often utilized for the purposes of regression and classification tasks. The KNN algorithm is a kind of instance-based learner, commonly referred to as idle learning. It does not build a categorization model-based approach till it is given samples to classify. The fundamental premise of KNN in categorization is to compare individual test samples with k nearby training samples in the variable space. The category of the test sample is determined based on the classification of its nearest k neighbors. Neighbors are often determined by calculating the Euclidean distance between the data point being analyzed and its k nearest neighbors. The k parameter, denoting the quantity of nearest neighbors’ number, is often kept minimal to avoid the inclusion of excessive data points that may distort the underlying characteristics of the data point under consideration. It is important to choose acceptable values for k in order to avoid overfitting and model instability, since large values of k might contribute to both issues. KNN utilizes the Euclidean distance metric. The underlying assumption is that each element in the dataset may be shown as a point in a space with N dimensions. KNN utilizes a parameter k to denote the number of examples to be considered, based on which the majority class is selected to categorize the new instance.


(3) $$E_{i}=\sqrt{\left(x_{1}-x_{2}\right)+{\left(x_{3}-x_{4}\right)}^{2}}$$

where $x_{1}$ , $x_{2}$ , $x_{3}$ , and $x_{14}$ calculate of the Euclidean distance in a two-dimensional space.

#### 3.3.2 Decision tree

A decision tree (DT) is a well-recognized nonparametric supervised learning technique. DT is one of the ML algorithms that can be applied for both regression and classification tasks. DT classifies the instances by traversing down the tree from the root to certain leaf nodes. Instances are categorized by evaluating the attribute specified by the node, beginning at the root node of the tree, and thereafter down the tree branch associated with the attribute value. The most often used criteria for splitting are “gini” for measuring Gini impurity and “entropy” for quantifying information gain, which may be mathematically represented.


(4) $$\mathrm{Entropy}=\left(S\right)=\overset{C}{\underset{i=1}{\sum }}p_{i}\;\log_{2}\;p_{i}$$

(5) $$\mathrm{Entropy}\;\left(S\;|B\right)=\overset{j}{\underset{j=1}{\sum }}\frac{\left|s_{i}\right|}{\left|S_{i}\right|}\;\mathrm{entropy}\;\left(S_{i}\right)$$

(6) $$\mathrm{Gain}\;\left(S\;|B\right)=\mathrm{entropy}\left(S\right)-\mathrm{entropy}\left(S\;|B\right)$$

The training dataset is indicated as S, while the freezing of gait dataset is represented by the class c , which encompasses both attack and normal data. The likelihood of seeing data that belongs to class $S_{i}$ is represented as $P_{i}$ . This probability is specifically related to the subsets of class $S_{i}$ in the characteristics B.

#### 3.3.3 Random Forest

A random forest (RF) classifier is a well-recognized collaborative classification technique used in machine learning and data science across several application domains. This approach employs “parallel ensembling,” whereby several DT classifiers are concurrently trained on distinct sub-samples of the dataset. The ultimate result is decided via mainstream vote or averaging of the outcomes. Therefore, it reduces the issue of over-fitting and enhances both the accuracy of predictions and control. Hence, the RF learning model, which utilizes many decision trees, often exhibits higher accuracy compared to a model based on a single decision tree. In order to construct a sequence of decision trees with regulated diversity, the method associates bootstrap combination (bagging) with arbitrary attributes selection. It is versatile for both classification and regression issues and is suitable for both categorical and continuous variables. Table 4 shows parameters of RF model.

**Table 4:** RF parameters.

Parameters name	Values
Estimators	500
Criterion	gini
Min_samples_leaf	1
Max_depth	10
Max_features	auto
Random_state	42

#### 3.3.4 LightGBM approach

LightGBM approach is a gradient boosting context that employs tree-based learning techniques. It is specifically engineered to be widely spread and highly effective, offering the following benefits: Enhanced training velocity and increased efficacy; Reduced memory consumption LightGBM provides support for parallel and GPU learning; Proficient at managing enormous volumes of data LightGBM is a rapid, circulated, and efficient gradient-boosting system that relies on decision tree methods. It is extensively used in a range of machine-learning tasks, including regression, ranking, and categorization (55). It is a furthering method that utilizes numerous weak machine-learning methods to create a powerful learning model. Boosting methods amplify the weights of incorrectly classified data while reducing the weightiness of successfully categorized data. Table 5 shows LightGBM parameter.

**Table 5:** LightGBM parameters.

Parameters name	Values
Estimators	500
Learning_rate	0.01
Max_depth	10
Random_state	42

### 3.4 Gated recurrent unit–transformers

#### 3.4.1 Gated recurrent unit

The GRU is a fundamental architecture of recurrent neural networks (RNNs) that has resemblance to Long Short-Term Memory (LSTM) models. GRU is specifically developed to represent sequential data by enabling the selective retention or loss of information over time. Nevertheless, GRU possesses a more streamlined structure compared to LSTM, with a reduced number of parameters. This characteristic facilitates training and enhances computing efficiency.

The GRU is designed to handle sequential data by iteratively updating its hidden state in response to both the current input and the prior hidden state. During each iteration, the GRU calculates a “candidate activation vector” that integrates data from the input and the preceding hidden state. Subsequently, the candidate vector is employed to modify the concealed state for the subsequent time step. Two gates, namely the reset gate and the update gate, are used to calculate the candidate activation vector. The reset gate is responsible for determining the extent to which the previous hidden state is disregarded, whereas the update gate is responsible for determining the extent to which the candidate activation vector is integrated into the future hidden state.


(7) $$\mu_{t}=\sigma \left(V_{\mu }x_{t}+W_{\mu }o_{t-1}+b_{\mu }\right)$$

(8) $$r_{t}=\sigma \left(V_{r}x_{t}+W_{r}o_{t-1}+b_{\mu }\right)$$

(9) $$i_{t}=\tanh\;\left(V_{o}x_{t}+W_{o}\left(r_{t}⊙o_{t-1}\right)+b_{0}\right)$$

(10) $$o_{t}=\sigma (\mu_{t}⊙o_{t-1}\;\left(1-\mu_{t}\right)⊙i_{t}$$

**Figure 6:**
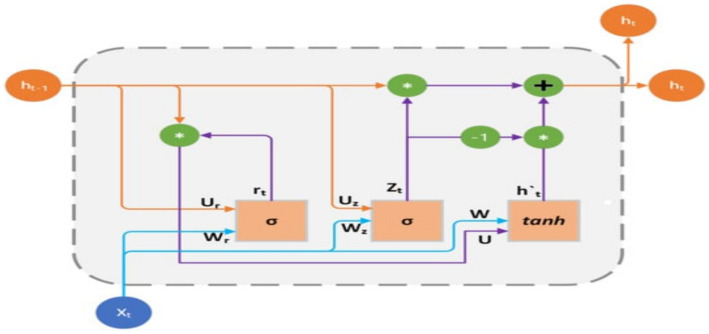
GRU structure.

Input is $i_{t}$ , output is $o_{t}$ , update gate output is $\mu_{t}$ , reset gate output is $r_{t}$ , and Hadamard product is ⊙. Weight matrices V, W, and b are parameters. The GRU encoder and Transformer path embeds input sequences using a recurrent GRU layer. Thirty-two GRU units encoded 200-dimensional vectors each timestep. Using multi-head self-attention with two heads, GRU embeddings may attend to each other based on learnt connections. Residual connections and layer normalization stabilize training. Flattening attention outputs to 1D vectors. Structure of GRU mode is presented in Figure 6.

#### 3.4.2 Transformers

The self-attention mechanism-based sequence-to-sequence model Transformer is extensively used in natural language processing methods including machine translation, text summarization, language synthesis. Significant outcomes are achieved quickly. Transformers has a different architecture than RNN. The Transformer branch in the proposed GRU-Transformer model assumes a crucial function in capturing complex interdependencies and multidimensional characteristics present in the input sequence. The aforementioned objective is accomplished by utilizing the self-attention and multi-head attention processes of the Transformer, as seen in Figure 3. Its attention-based encoder-decoder structure enables the Transformer to effectively handle sequence-to-sequence tasks.


(11) $$Q={XW}_{Q}$$

(12) $$K=XW_{K}$$

(13) $$V=XW_{V}$$

(14) $$A=\mathrm{Softmax}\left(\frac{QK^{T}}{\sqrt{d_{k}}}\right)$$

(15) Y=AV

Where, X be the input and (K, Q, 𝑉) is query matrix, key matrix, value matrix, learnable weight matrix is A , attention matrix is Y , output matrix is $\sqrt{d_{k}}$ , and attention header dimension, the scaling factor, reduces overly large or minuscule attention weights. To determine key value weight, softmax is used as a normalizer. The attention mechanism calculates the association between each input sequence item and the others to capture global dependencies.

The unit recurrent layer is 200 unit that stores sequence data and may capture dependencies. The parameter “return_sequences” sends the sequence of outputs for each time step to the next layer instead of just the final output. This Transformer component lets the model focus on different input sequence segments during prediction. Two 200-key dimension attention heads are used in the suggested method. This implementation helps the layer capture data relationships and connections. Attention boosts and accelerates learning. The residual link, or skip connection, solves the fading gradient problem by offering an alternate gradient movement path. Each time step of the sequence receives an individual 120-unit dense layer to extract unique characteristics. This strategy stochastically assigns input units to 0 during training after the TimeDistributed layer at 0.2 to reduce overfitting.

**Figure 7:**
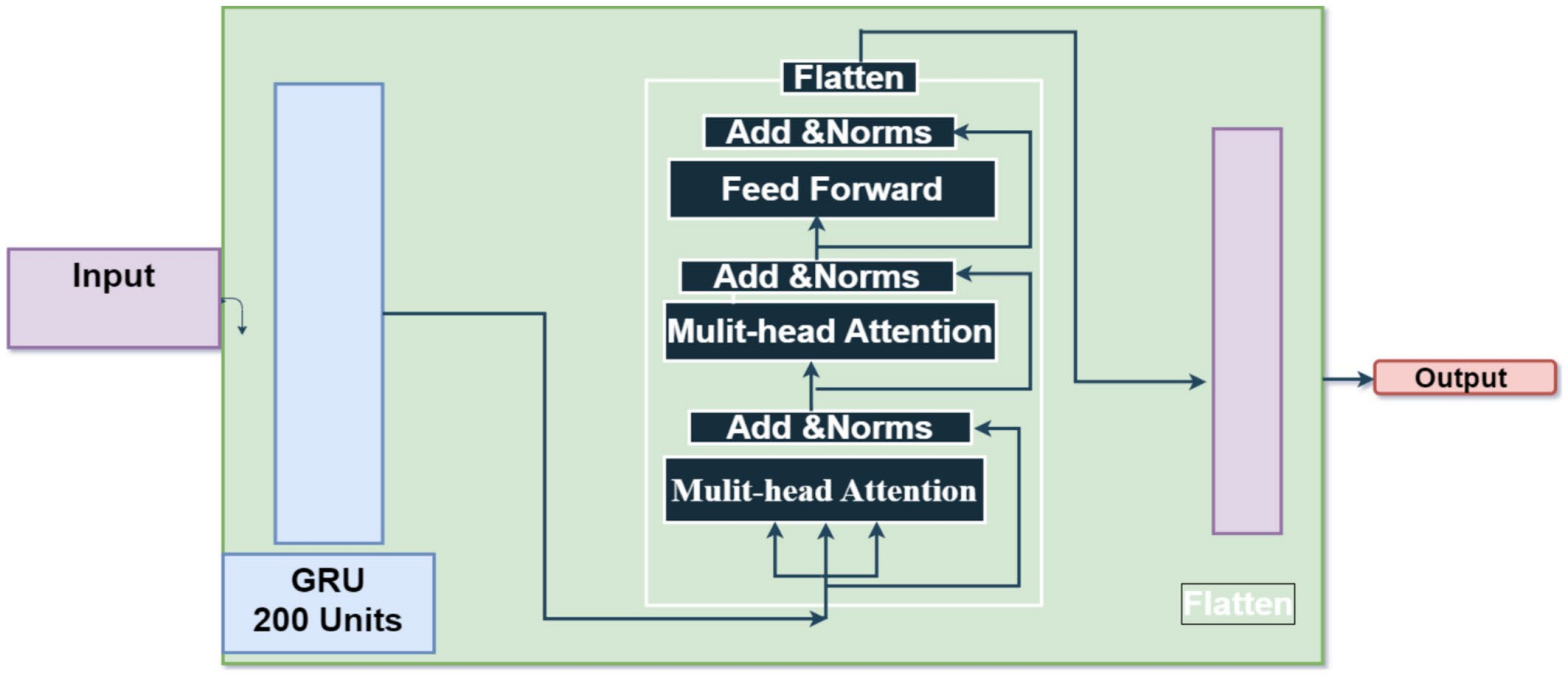
GRU-transformers.

The output of the previous layers is turned into a unified vector to link with the final Dense layer for classification. The neural network generates probabilities for each of the four classes using a Dense layer with softmax activation. Figure 7 shows the structure of GRU-transformers. Parameters of GRU-transformers is presented in Table 6.

**Table 6:** paramters of GRU-transformers.

Parameters	Values
GRU	32 units
Multi-Head Attention	2 heads
Add	2 heads
Layer Norm	--
Flatten	---
Dropout	0.5
Dense	1,024

### 3.5 Long-term recurrent convolutional networks

LRCN neural networks combine the strengths of the CNN and RNN to handle sequential input with spatial and temporal dependency. The model’s early layers use Convolutional Neural Networks (CNNs) to extract spatial properties from input data. These collected characteristics feed Recurrent Neural Networks (RNNs) to capture temporal relationships and long-term correlations. LRCN may acquire spatial and temporal complex data representations by integrating CNN and LSTM components. This neural network design handles sequential data well. LRCN is an RNN developed to evaluate its performance on sequence input data.


(16) $$C=\overset{i}{\underset{1}{\sum }}\overset{j}{\underset{1}{\sum }}I_{ij}F_{ij}$$

(17) $$C_{i}^{L}=B_{i}^{L}+\overset{x^{\left(L-1\right)}}{\underset{j=1}{\sum }}F_{i,j}^{L}*C_{j}^{\left(L-1\right)}$$

Where, F represents a convolution kernel or filter, while i and j represent rows and columns of dataset. A unique two-dimensional output is obtained by convolving the input dataset.

With the kernel. $B_{i}^{L}$ represents the bias matrix, whereas $F_{i,j}^{L}$ represents the filter connecting the jth feature map in the layer.


(18) $$f_{t}=\sigma (W_{ef}X_{t}+W_{ef}h_{t-1}+W_{cf}C_{t-1}U_{f}\;+)$$

(19) $$i_{t}=\sigma (W_{\mathrm{xi}}X_{t}+W_{hi}h_{t-1}+W_{ci}C_{t-1}+U_{i}$$)

(20) $$C_{t}=\sigma (f_{t}c_{t-1}+i_{t}\tanh(W_{xc}X_{t}+W_{hc}h_{t-1}+U$$)

(21) $$o_{t}=\sigma \left(W_{xo}X_{t}+W_{ho}h_{t-1}+W_{co}C_{t-1}+U_{o}\right)\text{,}$$

(22) $$h_{t}=O_{t}\times \tanh(C_{t}$$)

**Figure 8:**
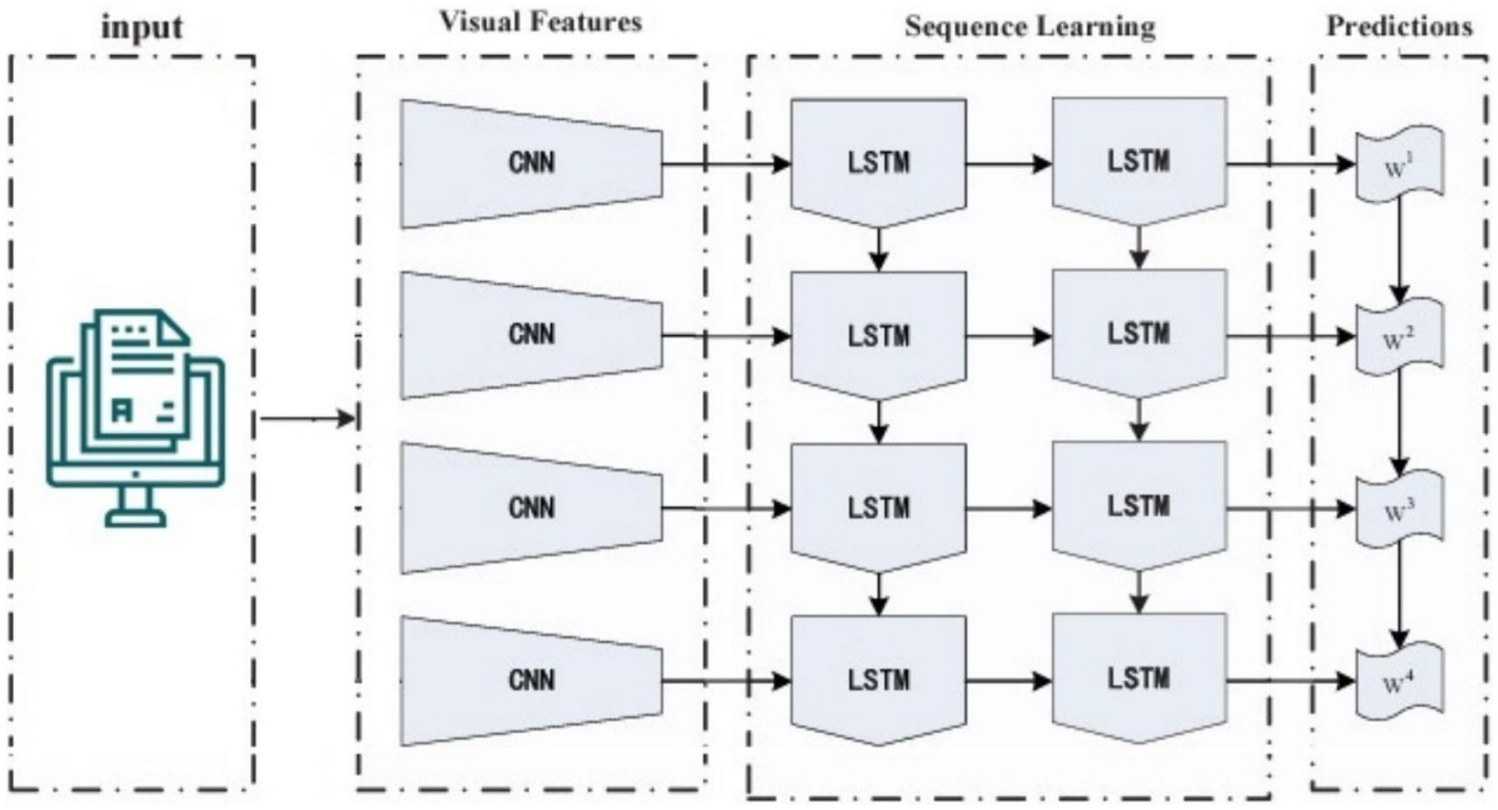
Structure of LRCN model.

Sequential forward and reverse methods apply the equations above. They represent the LSTM model equations. A gated cell in the LSTM network evaluates input data and retains it based on relevance or weight. The input gate, forget gate, and output gate make up the LSTM model. The forget gate $f_{t}$ decides whether states to keep or discard. The input gate $i_{t}$ modifies the value based on signals. The output gate $o_{t}$ transmits cell status to neighboring neurons. The design has a logistic layer and a layer that generates a new vector to mix with the state. In a recurrent neural network (RNN), the hidden layer processes $X_{t}$ using the weight matrix W to produce yt. The LSTM model uses a memory cell called $h_{t}$ , which is governed by three gates. The structure of LRCN is presented in Figure 8.

### 3.6 Evaluation metrics

Prior to further exploring our study, it is essential to elucidate the significance and computation techniques of several assessment measures. In this study, we have selected four primary assessment metrics: accuracy, precision, recall, f1-score, and rate of change (ROC).


(23) $$\mathrm{Accuracy}=\frac{TP+TN}{TP+FP+FN+TN}\times 100\%$$

(24) $$\mathrm{Recall}=\frac{TP}{TP+FN}\times 100\%$$

(25) $$\mathrm{Precision}=\frac{TP}{TP+FP}\times 100\%$$

(26) $$\mathrm{Fscore}=\frac{2*\mathrm{preision}*\mathrm{Sensitivity}}{\mathrm{preision}+\mathrm{Sensitivity}}\times 100\%$$

Algorithms of ML algorithms.

Let D be the dataset containing sensor data from FoG Parkinson’s disease patients, where *D = {(X_i,_ Y_i_)} ^N^_i = 1_* where *X_i_* represents the features and *Y_i_* represents the corresponding FoG labels.

*D* is collected from wearable devices.

*Data preprocessing.*

clean the data $D^{\prime }=\mathrm{clean}\;\left(D\right)\text{.}$

Normalize the data $D^{\prime \prime }=\mathrm{normalize}\;\left(D^{\prime }\right)$

Resample the data $D^{\prime \prime \prime }=\mathrm{resample}\;\left(D^{\prime \prime }\right)$

Feature extraction

Extract features: *X = {X_i_} ^N^_i = 1_*

Model training

4.1 Select machine learning algorithms: ML_Algorithms = {DT,RF, KNN, LightGMBet, CatBoost}

4.2 Split the data into training and testing sets: $D_{\mathrm{train}},D_{\mathrm{test}}=\mathrm{Split}\;\left(D^{\prime \prime \prime },70\%\right)$

4.3 Train the models: $\mathrm{Mode}l_{j}=\mathrm{train}\;\left(M{L_{\mathrm{algorithm}}}_{j},D_{\mathrm{train}}\right)\text{;}$ *j = 1,2,3, … num_algorithms*

*Model evaluation*

*evaluate models _j_: Metrics _j_ = evaluate (* $\mathrm{Mode}l_{j},D_{\mathrm{test}}),j=1,2,3,\ldots num:\mathrm{algorithms}$

*FoG Detection:*

*Predict FoG instances: Ῠ = predict (* $\mathrm{mode}l_{\mathrm{best}},X)$

*FoG_Events = detect (Ῠ)*

## 4 Experimental

This section presents the classification results and discoveries derived from a sequence of experiments carried out for predicting PD FoG by applying machine-learning algorithms. The main aim of these experiments was to evaluate the efficacy of several classification models in accurately distinguishing various types of classes associated with gait behavior, specifically Normal, Turn, StartHesitation, and Walking. The evaluation primarily examined evaluation parameters such as accuracy, precision, recall, and f1-score for each class, offering valuable insights into the capabilities as well as limitations of the applied models. This part included simulation setup, split dataset, and machine-leaning results.

### 4.1 Simulation setup

This module encompasses the specific steps and procedures involved in carrying out our suggested approaches. The instruments used in this document are enumerated in Table 7.

**Table 7:** Environmental requirements of the presented model.

Hardware	Software
RAM size 16 GB Intel(R) Core(TM) i7 CPU GHz	Python Panda TensorFlow library Keras library Matplotlib NumPy library

### 4.2 Split dataset

The dataset was divided into a 70% training dataset and a 30% testing dataset.

### 4.3 Results

#### 4.3.1 Random forest testing results

Table 8 provides the testing results of the RF model for PD FoG. It had strong performance in accurately differentiating the “Turn class,” with a precision of 0.98, recall of 0.99, f1-score of 0.96, and a total accuracy of 90%. Though, there were complications in precisely detecting occurrences of the Turn class, as the recall rate was significantly lower despite a high precision score.

**Table 8:** Testing results of the RF model.

Model	Class	Precision %	Recall %	F1-score %	Accuracy %
RF	Normal	87	85	90	90
	startHesitation	98	99	96	
	Turn	90	91	89	
	Walking	94	96	93	
	Weighted	90	90	90	

**Figure 9:**
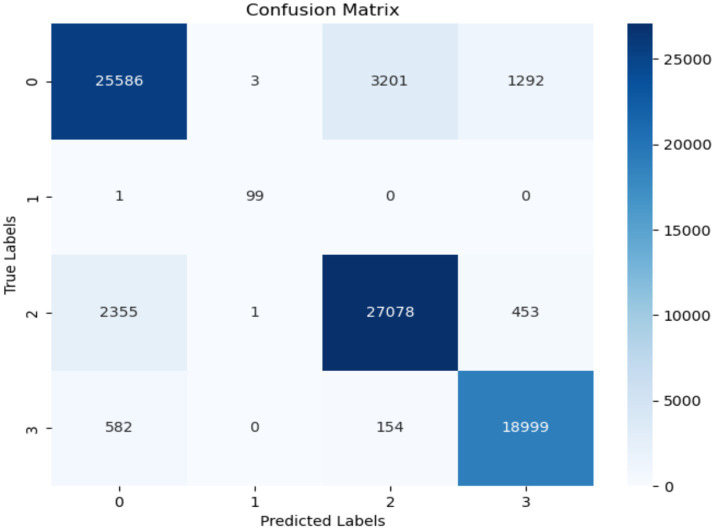
Confusion matrix of RF.

Figure 9 displays the confusion matrix of the RF model used for the classification of FoG of PD disease patients. The misclassification rate of the RF model in diagnosing FoG is less. The RF model exhibited a true negative rate of 25,586 for the classification of FoG. The number of true positive instances classified are 25,586 as Normal, 99 as Turn, 27,078 as startHesitation, and 18,999 as Walking.

#### 4.3.2 Decision tree testing results

The experimental results while using the DT model demonstrated exceptional and excellent performance, notably in accurately categorizing the instances labeled as Turn. The model demonstrated exceptional precision (97%), recall (99%), F1-score (94%), and an overall accuracy of 91% for classes that existed in the clinical experimental dataset used. Although the model demonstrated strong accuracy and recall overall, it encountered difficulties in accurately detecting instances of the Turn class. This is evident from the poorer precision and recall scores specifically associated with this class. Table 9 summarizes the classification results based on the DT model.

**Table 9:** Testing results of the DT model.

Model	Class	Precision %	Recall %	F1-score %	Accuracy %
RF	Normal	89	89	89	91
	startHesitation	97	99	94	
	Turn	90	90	90	
	Walking	96	97	95	
	Weighted	91	91	91	

**Figure 10:**
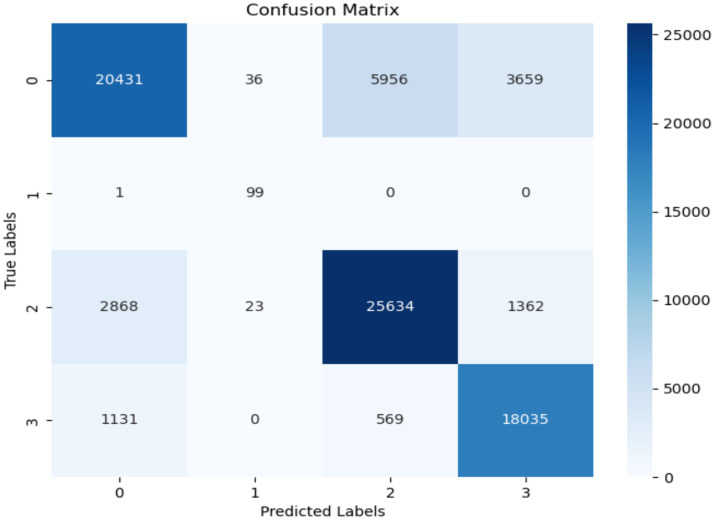
Confusion matrix of decision tree.

The confusion matrix in Figure 10 displays the performance of the decision tree approach. The decision tree algorithm achieved a high accuracy of 91% throughout an evaluation stage. The program accurately classified 20,431 instances as normal. The misclassification of the class startHesitation is 2,868 instances more than that of the other classes, while the misclassification of the class Turn is only 1 instance.

### 4.4 K-nearest neighbor’s classification results

The KNN model had excellent performance in accurately identifying instances belonging to the Walking class, achieving high precision (73%), recall (82%), f1-score (66%), and a total accuracy of 63%. Nevertheless, there were notable limitations in effectively classifying the Turn class samples, with both precision and recall scores being significantly noted in testing classification reports. Table 10 demonstrates the classification results based on the KNN model (Table 10).

**Table 10:** Testing results of the KNN model.

Model	Class	Precision %	Recall %	F1-score %	Accuracy %
RF	Normal	58	53	64	63
	startHesitation	63	1.00	47	
	Turn	61	61	61	
	Walking	73	82	66	
	Weighted	63	63	63	

**Figure 11:**
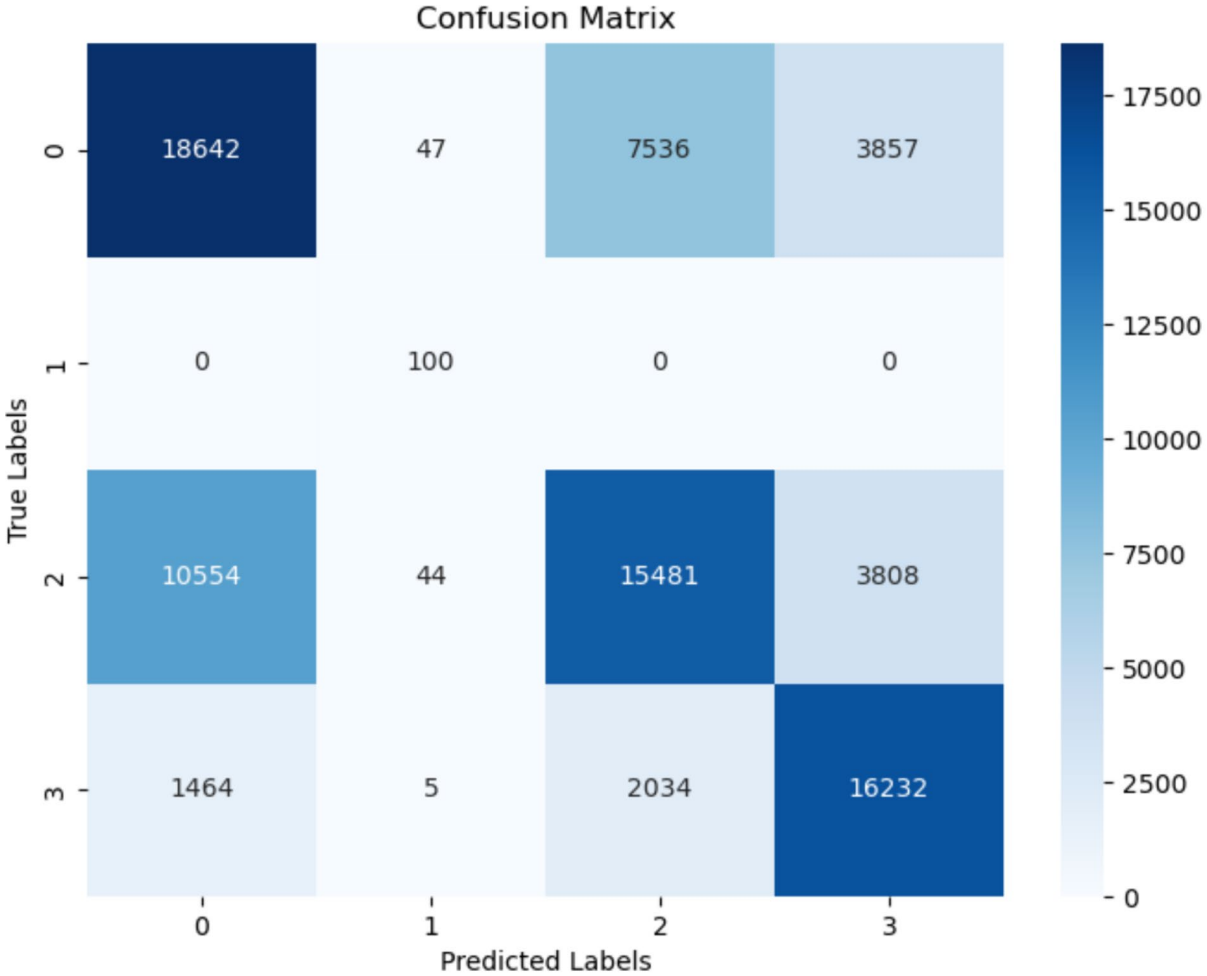
Confusion matrix of KNN.

The confusion matrix for the KNN model is displayed in Figure 11. The number of instances correctly predicted as “Normal” is 18,642, whereas there are no instances incorrectly predicted as “Turn.” However, the false positive rate is significantly high. The rate of false positives for the “startHesitation” class is particularly high, with a value of 10,554.

### 4.5 Classification results using the LightGBM model

This subsection presents the findings in detail of the classification results of the LightGBM model, which exhibited significant precision (84%), recall (91%), f1-score (78%), and overall accuracy (80%) in accurately categorizing the “Walking” cases. We faced complications in accurately identifying instances of the “Normal” category, leading to lower precision and recall scores. Table 11 displays the testing results of the LightGBM model.

**Table 11:** Testing results of the LightGBM model.

Model	Class	Precision %	Recall %	F1-score %	Accuracy %
LightGBM	Normal	75	68	84	80
	startHesitation	77	99	63	
	Turn	83	86	80	
	Walking	84	91	78	
	Weighted	81	80	80	

**Figure 12:**
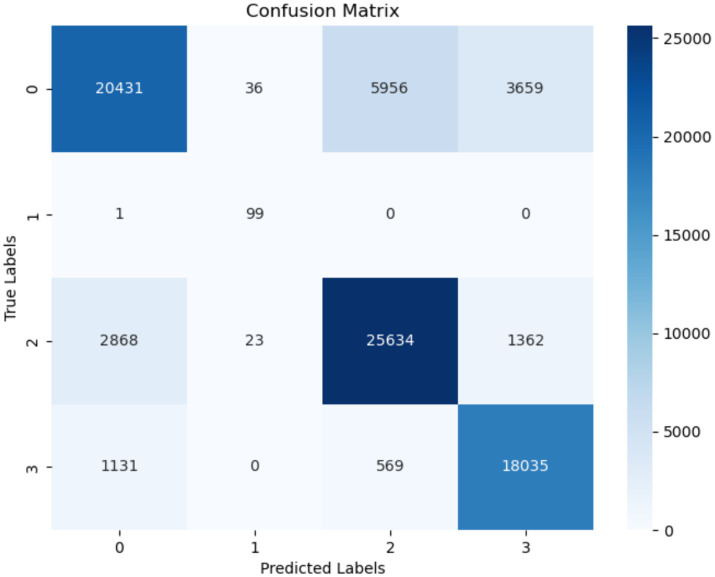
Confusion matrix of LightGBM.

Figure 12 displays the confusion matrix of the LightGBM model. It is worth noting that the misclassification (FP) rate for the “startHesitation” class is significantly high, with a total of 2,868 instances. The occurrence of false positives in the “Turn” class is extremely low, less than 1. The number of instances correctly classed as “Normal” and identified as negative is 20,431.

### 4.6 CatBoost model classification results

**Figure 13:**
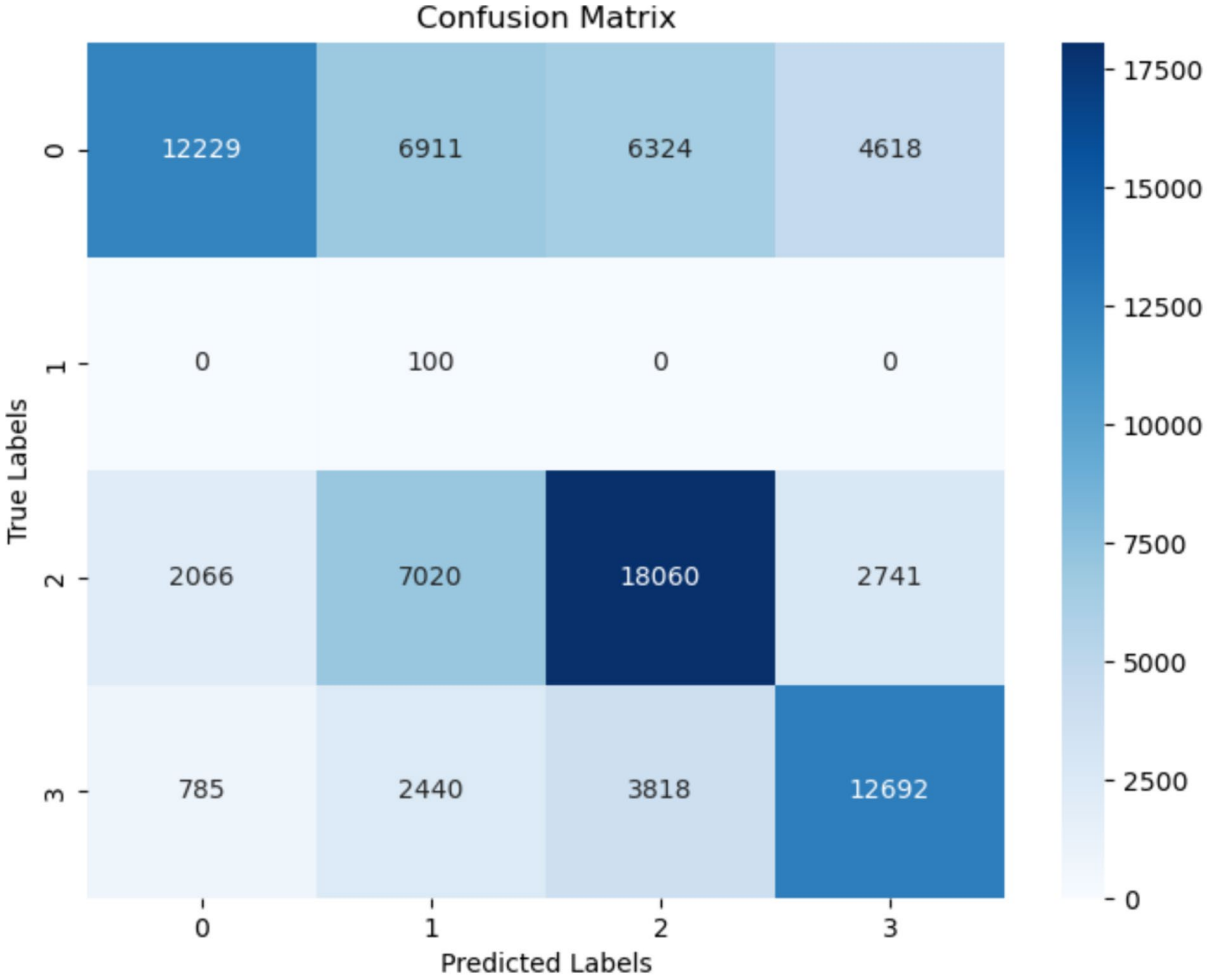
Confusion matrix of CatBoost.

This section presents the results of the CatBoost model. The CatBoost algorithm exhibited remarkable precision (80%), recall (92%), f1-score (86%), and overall accuracy (82%) for the “Walking” class. Nevertheless, there were limitations in accurately categorizing cases that fell within the “startHesitation” class, leading to relatively low precision and recall ratings. Table 12 presents the testing and classification outcomes of the CatBoost model. The confusion matrix of CatBoost is presented Figure 13.

**Table 12:** Testing results of the CatBoost model.

Model	Class	Precision %	Recall %	F1-score %	Accuracy %
CatBoost	Normal	85	71	77	82
	startHesitation	27	1.00	42	
	Turn	81	86	84	
	Walking	80	92	86	
	Weighted	82	82	82	

### 4.7 Results of GRU-transformers and LRCNN models

In this section GRU mode was combined with transformers model for classification FoG, we have used 200 hidden units for GRU model. Table 13 shows the parameters of GRU-transformers and LRCNN models. It is noted that the accuracy of GRU-transformers and LRCN were achieved. It is investigated that the GRU-transformers and LRCN were better models for classification FoG.

**Table 13:** Weight Avg. results of GRU-transformers and LRCNN model.

Models	Accuracy %	Precision%	Recall %	f1-score %
GRU-transformers	86	84	86	83
LRCNN	86	85	86	84

**Figure 14:**
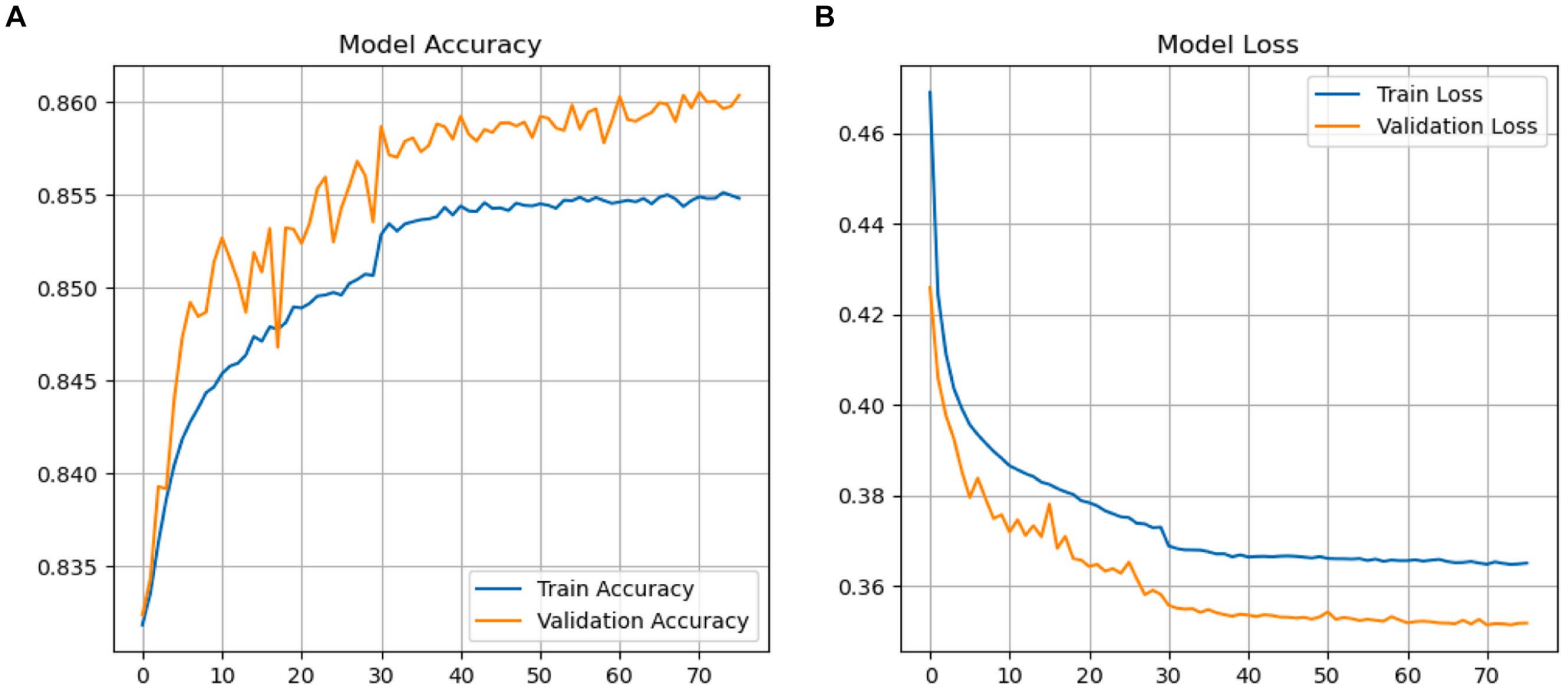
**(A,B)** Performance GRU-transformers.

The accuracy performance of the GRU-transformers is depicted in Figure 14. The GRU-transformers validation accuracy initially stood at 82% and then improved to 86% after 70 Epochs. The accuracy loss started from 0.43 and reached 0.32.

**Figure 15:**
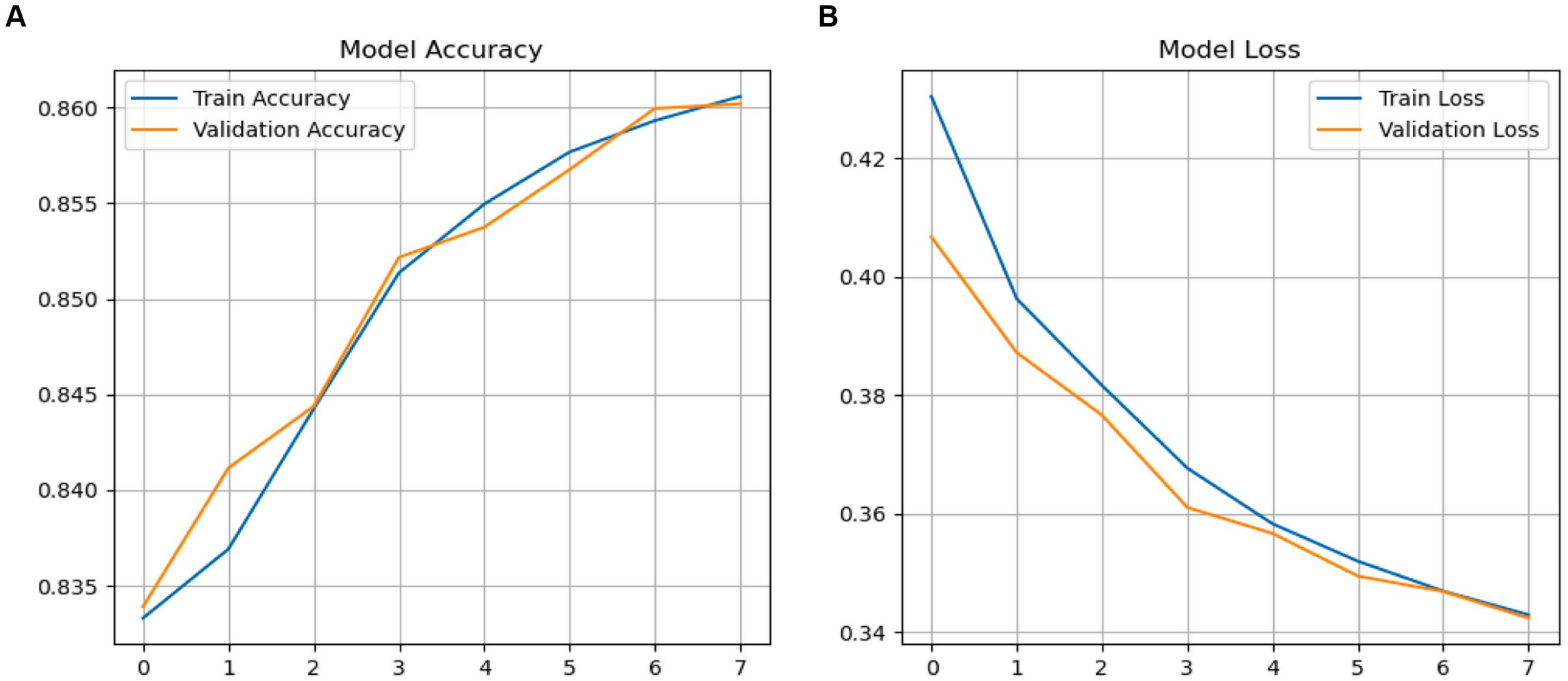
**(A,B)** Performance LRCN model.

The performance and loss accuracy in the validation stages was calculated using the binary_crossentropy approach. The validation accuracy of the LRCN model is depicted in Figure 15. During the validation phase, the LRCN model exhibited started at 38% and reached to 86%. The accuracy loss is a decrease in accuracy loss from 0.42 to 0.35.

## 5 Results discussion

FoG is a motor disturbance categorized by an abrupt and fleeting inability to start or maintain walking, which poses difficulties for patients with PD. The timely identification and predicting of FoG episodes are essential for efficient therapies and enhanced quality of life. The objective of this research was to evaluate the possibility of applying different machine-learning algorithms and GRU-transformers and LRCN models to predict FoG for a preventive strategy to mitigate the occurrence. In order to achieve this objective, random forest, k-nearest neighbor, LightGBM, and GRU-transformers and LRCN models algorithms were applied for detecting FoG.

**Figure 16:**
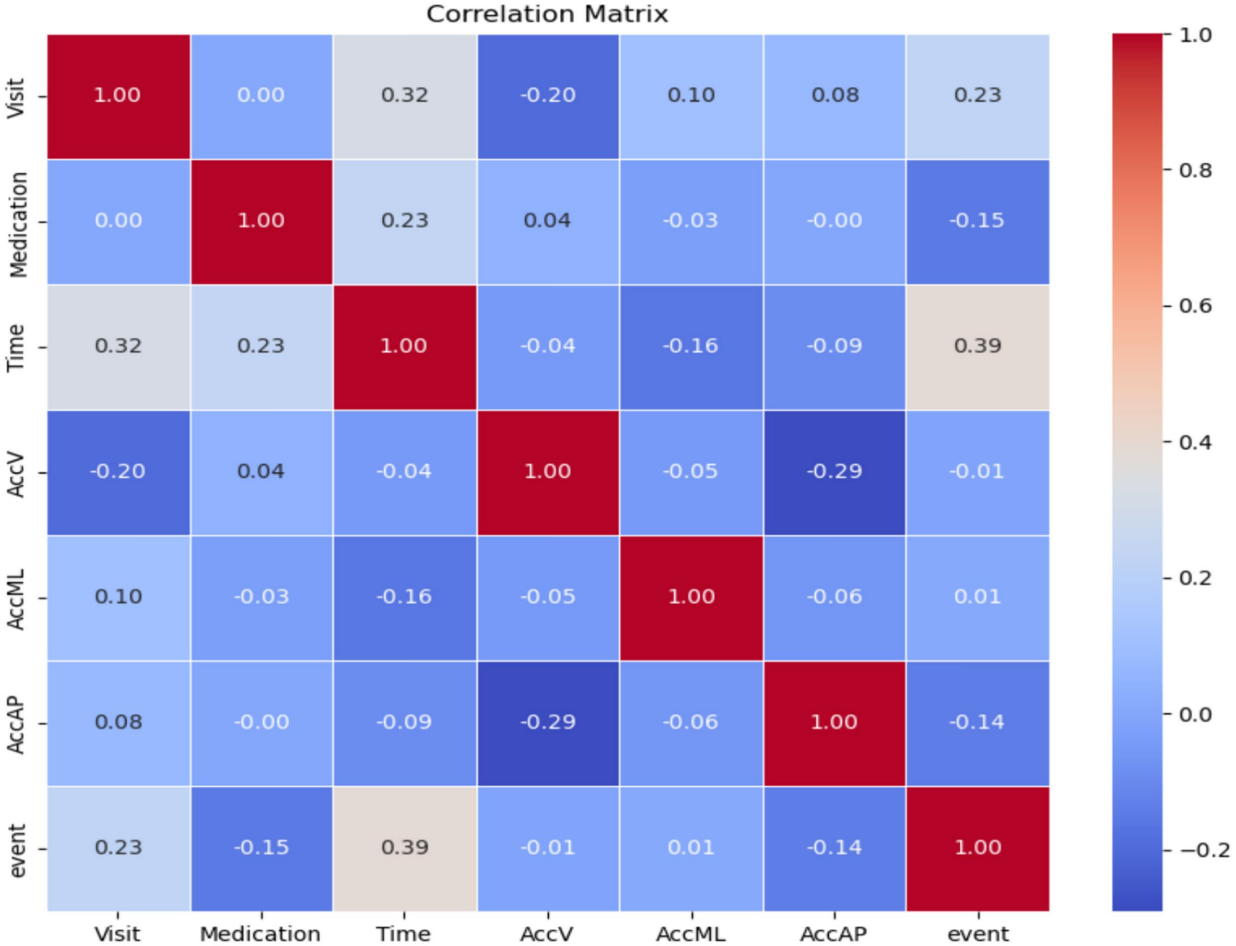
Correlation between features of the training dataset.

The difficulties in classifying minority classes, specifically “startHesitation,” highlight the influence of imbalanced datasets on the effectiveness of models. Addressing these problems is essential in the context of FoG prediction to enable early detection of gait irregularities, facilitate prompt interventions, and enhance outcomes for individuals with PD. Therefore, we have applied an oversampling method for handling the imbalanced classes at the training phase. Figure 16 the relationship among features of the training dataset.

According to the experimental results obtained from using various machine-learning models, the decision tree model had a strong overall accuracy of 91% and an impressive f1-score of 0.96, particularly excelling in accurately categorizing “Normal” gait. Nevertheless, the difficulties in precisely recognizing occurrences of “Turn” highlight the necessity of adjusting and optimizing the detection process to achieve a balance between accuracy and comprehensiveness. This is crucial for reliably identifying tiny irregularities in walking patterns that indicate FoG. The decision tree model demonstrated a notable weighted accuracy of 91% for all classes. The random forest approach scored a high accuracy (90%). The KNN algorithm demonstrated a commendable level of accuracy (63%) and precision (63%). However, it is noted that the KNN achieved less accuracy compared with different existing ML approaches. Comprehending the influence of distance metrics and the quantity of neighbors is essential for enhancing its capacity to detect tiny variations linked to FoG. The LightGBM model showed potential, specifically in accuracy, attaining an accuracy of 80% and an f1-score of 0.80. The CatBoost model demonstrated a strong precision of 0.82 and recall of 0.82, resulting in an accuracy of 82% and an f1-score of 82.

The ROC curve is a visual depiction that displays the performance of a classification algorithm at different levels of categorization. The graph depicts the relationship between two variables. The receiver operating characteristic (ROC) is computed using the following formula:


(24) $$TRP=\frac{TP}{TP+FN}$$

(25) $$FPR=\frac{FP}{FP+TN}$$

Where TRP is the true positive rate and FPR is the false positive rate.

**Figure 17:**
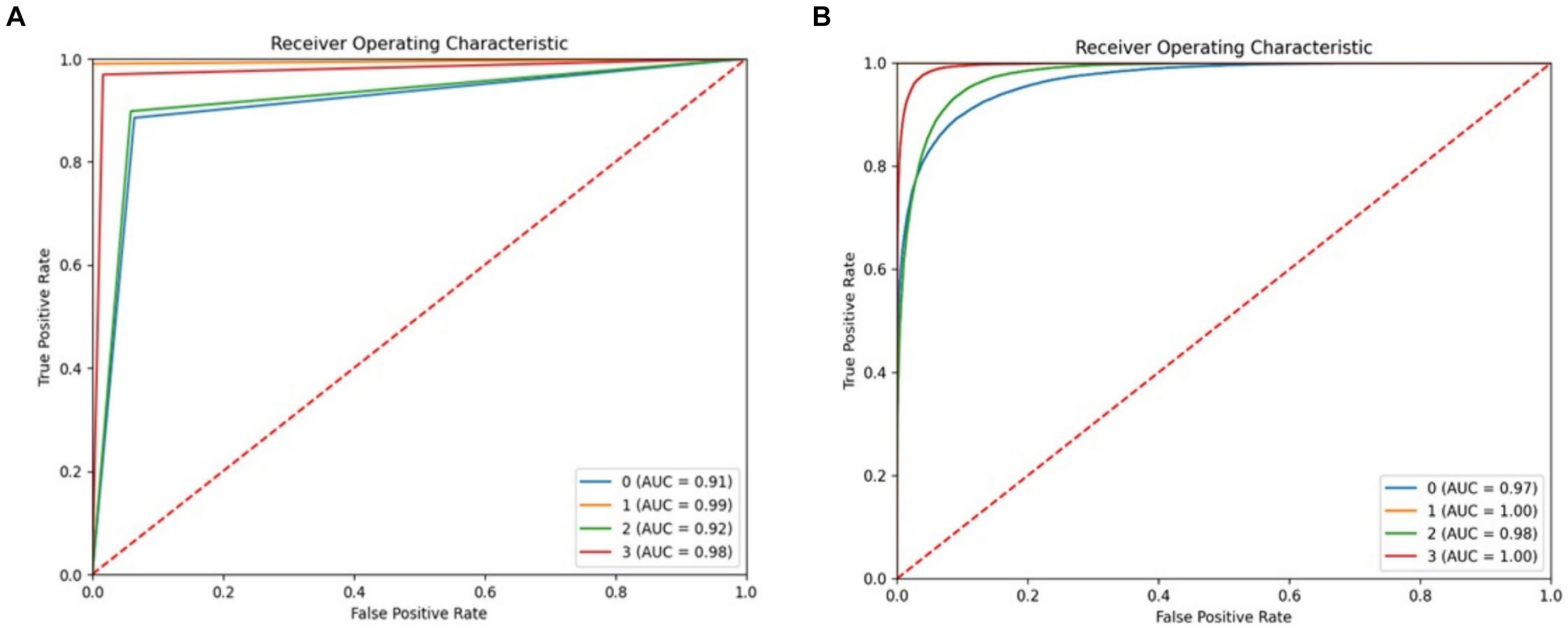
ROC of proposed system, **(A)** RF **(B)** decision tree.

Figure 17 displays the ROC curve for both the DT and RF models. The DT model achieved a high ROC score of 99% for the “startHesitation” class and an ROC score of 98 for the “Walking” class. The receiver operating characteristic (ROC) analysis of the RF model yielded a high precision of 100% for the “startHesitation” class and an ROC value of 98% for the “Turn” class.

**Figure 18:**
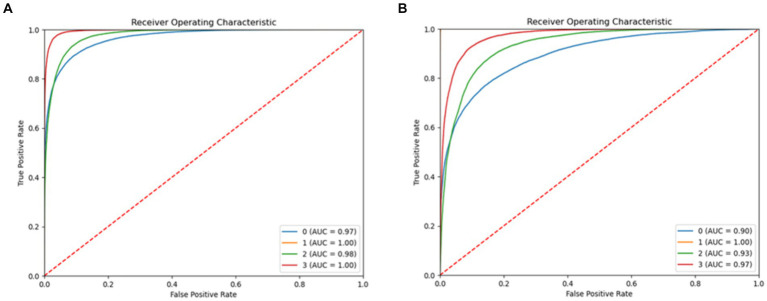
ROC of proposed system, **(A)** LightGBM **(B)** CatBoost.

The LightGBM, and CatBoost algorithms scored less in accuracy. However, the ROC of the models are good, and the LightGBM, and CatBoost models scored ROC 100% in the “startHesitation” class. Figure 18 displays the ROC of LightGBM, and CatBoost models.

**Figure 19:**
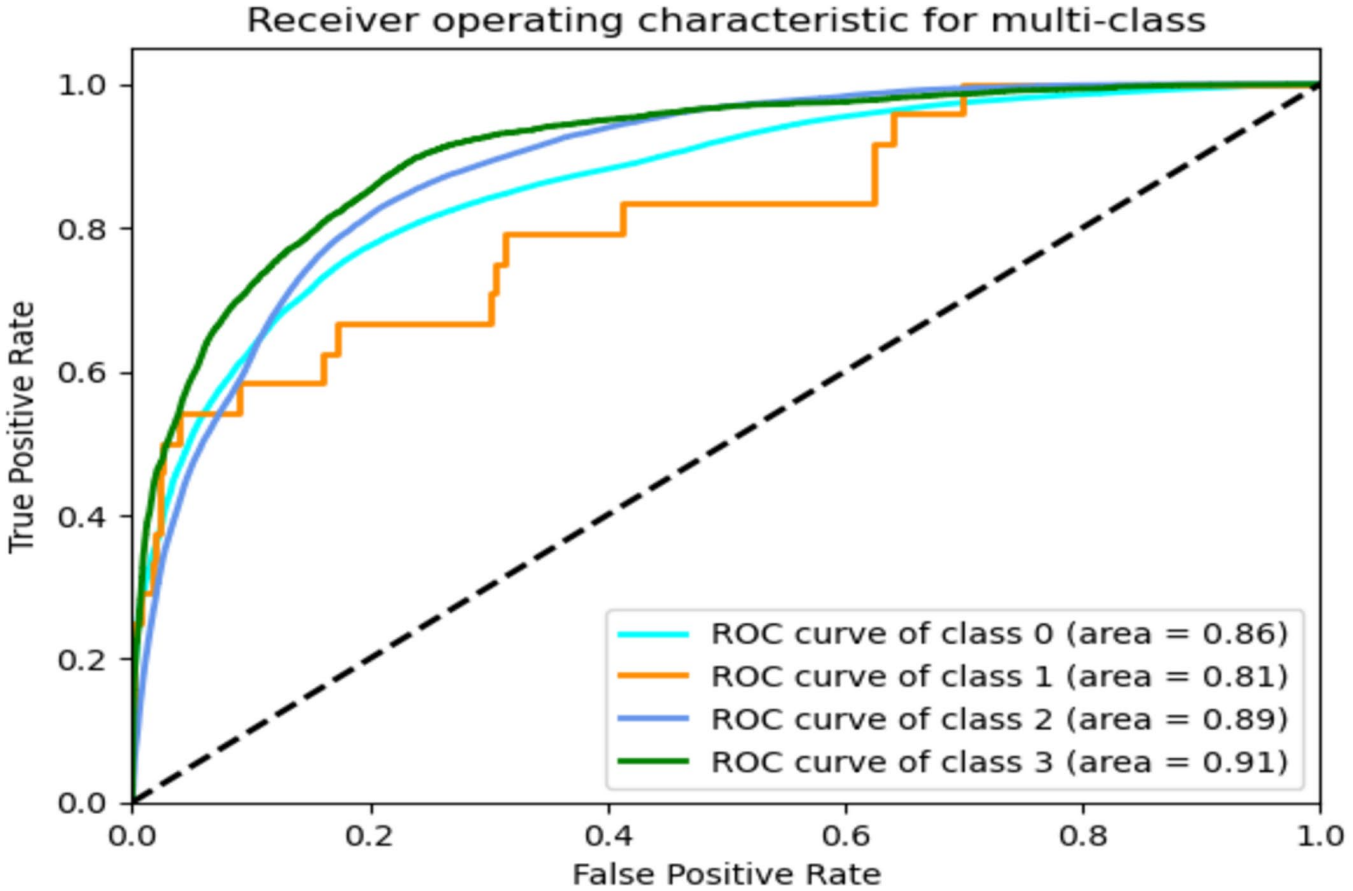
ROC of GRU-transformers.

**Figure 20:**
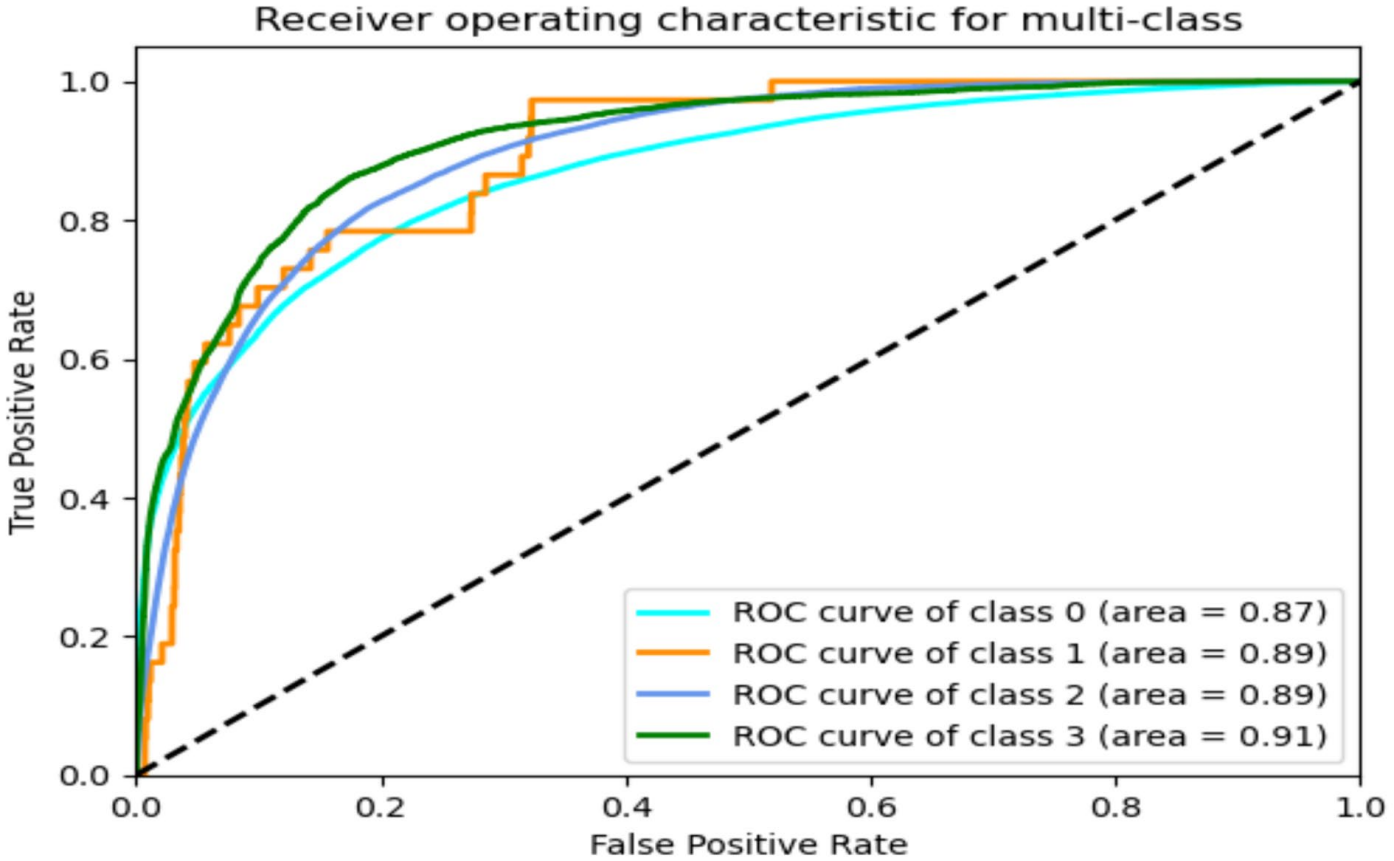
ROC of LRCN.

Figures 19, 20 diplays ROC of GRU-transformers and LRCN models for predicting FoG. It is noted both models have achieved similar performance, and GRU-transformers and LRCN were scored high percentage ROC = 91 with class “Walking”.

Table 14 presents a comparison of the suggested algorithms, highlighting that the decision tree technique achieved a high accuracy rate of 91%.

**Table 14:** Comparison results.

Models	Accuracy (%)
Random forest	90
Decision tree	91
KNN	63
LightGBM	83
CatBoos	80
GRU-transformers	86
GRU-transformers	86

## 6 Conclusion

FoG is a locomotive impairment observed in individuals with advanced PD, which has been linked to an elevated likelihood of falling and a worse overall quality of life. Freezing incidents can be alleviated or averted through external intervention, such as the utilization of pictorial or auditory signals, which are triggered by FoG detection and prediction systems. The fundamental aim of this research work was predicting FoG using a standard dataset. This research concerted on the detection and prediction of FoG by analyzing 3D accelerometer data collected from the lower back of persons with PD, who frequently experience this terrible symptom. The dataset was obtained from a cohort of 65 participants. The dataset consists of four distinct classes: Normal, Turn, startHesitation, and Walking. Preprocessing techniques, such as cleaning the dataset and addressing imbalanced classes, were implemented to enhance the performance of the ML methods. Various machine-learning algorithms, including decision tree, random forest, k-nearest neighbors, LightGBM, GRU-transformers and LRCN models, were employed for FoG detection and prediction. Of these, the decision tree algorithm exhibited a distinct advantage when working with datasets collected from sensors, achieving a high accuracy rate of 91%. This is the initial model employed for detecting FoG using this dataset. Furthermore, the main aim of this study also was to identify effective ML and DL algorithms that has capability of detecting and predicting FoG using a wearable system in real-time data.

## Data availability statement

Publicly available datasets were analyzed in this study. This data can be found here: https://kaggle.com/competitions/tlvmc-parkinsons-freezing-gait-prediction/data.

## Author contributions

AA-N: Writing – original draft, Methodology, Funding acquisition, Formal analysis, Data curation, Conceptualization. TA: Writing – review & editing, Software, Methodology, Funding acquisition, Formal analysis, Data curation, Conceptualization. NF: Writing – original draft, Visualization, Validation, Formal analysis, Data curation, Conceptualization. DK: Writing – review & editing, Visualization, Validation, Resources, Investigation, Funding acquisition, Formal analysis.
